# Comparative analysis of allele variation using allele frequencies according to sample size in Korean population

**DOI:** 10.1007/s13258-021-01159-z

**Published:** 2021-08-25

**Authors:** Hyun-Chul Park, Eu-Ree Ahn, Sang-Cheul Shin

**Affiliations:** Forensic DNA Division, National Forensic Service Daegu Institute, Daegu, 39872 Republic of Korea

**Keywords:** Allele frequency, Sample size, Sampling bias, Comparative study, Population study

## Abstract

**Background:**

Allele frequency using short tandem repeats (STRs) is used to calculate likelihood ratio for database match, to interpret DNA mixture and to estimate ethnic groups in forensic genetics. In Korea, three population studies for 23 STR loci have been conducted with different sample size for forensic purposes.

**Objective:**

We performed comparative analysis to determine how the difference of sample size affects the allele frequency and allele variation within same ethnic population (i.e. Korean). Furthermore, this study was conducted to check how the sampling group and multiplex kit also affect allele variation such as rare alleles and population specific alleles.

**Methods:**

To compare allele variation, we used allele frequencies of three population data published from three Korean forensic research groups. Allele frequencies were calculated using different sample sizes and multiplex kits: 526, 1000, and 2000 individuals, respectively.

**Results:**

The results showed the different distribution of allele frequencies in some loci. There was also a difference in the number of rare alleles observed by the sample size and sampling bias. In particular, an allele of 9.1 in the D2S441 locus was not observed in population study with 526 individuals due to multiplex kits.

**Conclusion:**

Because the allele frequencies play an important role in forensic genetics, even if the samples are derived from the same population, it is important to consider the effects of sample size, sampling bias, and selection of multiplex kits in population studies.

## Introduction

As short tandem repeat (STR) consists of 3–5 nucleotides repeat unit, it is located within introns and widely distributed on the genome. Even though each STR is not meaningful, the combination of STR on multiple loci has been used for individual identification in forensic genetic (Butler 2007). Allele at each locus is determined by the repeated number of STR. Since alleles for multiple loci are different for each person, it is used to identify the culprit or to confirm paternity.

Allele frequency refers to the relative frequency of alleles at a particular locus in a population. Because each ethnic group has a different allele frequency, it is possible to distinguish ethnic groups within a population based on dissimilarity of allele frequencies (Butler 2014). Particularly, in forensic genetics, the allele frequency is used to calculate various statistical probabilities, such as a random match probability and likelihood ratio for paternity testing, DNA mixture interpretation, and database for the evidence and the suspect’s DNA match. Furthermore, web-based platforms for predicting major population groups and the quality control of STR databases using allele frequencies have been constructed (Pereira et al. 2011; Bodner et al. 016). Population studies using STR for country and ethnic groups are consistently conducted with various sample size. Chakraborty (1992) reported that 100–150 individuals are the appropriate sample size to calculate allele frequency at variable number tandem repeat (VNTR). Depending on the number of samples used in a population study, the variation and frequency of allele can lead to different results, which affect statistical probability and data interpretation. Even within a single population, differences in allele frequency and rare alleles can be detected due to the sample size (Einum and Scarpetta 2004; Hill et al. 2013).

After the CODIS core loci number was expanded from 13 to 20 (Hares 2015), three population studies that included the expanded CODIS loci were conducted in Korea (Park et al. 2013, 2016; Kim et al. 2017). Although these population studies were performed within the same ethnic group (i.e., Korean), the sample size and sampling groups for analysis are different. In this study, we conducted a comparative study to determine how factors such as sample size and sampling group affect the results of population study. We compared allele frequencies of 23 STR loci including 20 CODIS core loci and three additional loci (i.e., Penta E, Penta D, and SE33). The results showed some differences in the number of observed rare alleles and allele frequencies in some loci according to sample size. In particular, a specific allele (9.1) at the D2S441 locus was not detected in the smallest sample size group. This result could be useful information to consider size, selection, and composition of sample for population study.

## Materials and methods

For allele frequencies and statistical parameter data, three population study data of Korean analyzed with 526, 1000, and 2000 individuals were used as a group A, group B, and group C, respectively (Park et al. 2013, 2016; Kim et al. 2017). Group A (526 individuals) and Group B (1000 individuals) are independent data set. And Group C (2000 individuals) is data including 1000 samples of Group B. Group A and group B investigated the variations in the 23 STR loci, whereas group C investigated 20 CODIS STR loci, excluding Penta E, Penta D, and SE33. The allele frequencies of Penta E, Penta D, and SE33 of group C were analyzed after requesting the relevant data from the authors. Boxplots were constructed for the maximum, minimum, and interquartile range (IQR) of the allele frequency for each locus using R (https://www.r-project.org/). Number of observed allele and rare allele were analyzed using Microsoft Excel. In this study, the rare allele was designated as a value under the minimum allele frequency (MAF).

## Results and discussion

A total of 349 alleles were observed in three population studies of Korean. The number of alleles observed in each group was 280, 305, and 342, whereas the number of alleles that does not detected was 69, 44, and 7, respectively. Larger sample sizes detected more alleles due to rare alleles. Generally, the MAF is calculated as MAF = 5/2 N (wherein *N* is the  number of individuals) (National Research Council 1996). We calculated the following MAF values for the three groups: 0.00475, 0.0025, and 0.0012, respectively. Larger sample sizes corresponded to more alleles with frequencies less than the MAF (Table 1).

**Table 1:** Comparison of observed alleles among three groups

	*N* = 526 (Group A)	*N* = 1000 (Group B)	*N* = 2000 (Group C)
Observed alleles	280	305	342
Number of ND^a^	69	44	7
MAF^b^	0.00475	0.0025	0.0012
Number of < MAF	67	73	83
PD^c^ in SE33	0.991	0.992	0.986
PE^d^ in SE33	0.918	0.894	0.834
Multiplex Kits	Identifiler, NGM, Powerplex16, Powerplex ES	GlobalFiler Powerplex Fusion	GlobalFiler Powerplex Fusion

^a^Not detected

^b^Minimum allele frequency

^c^Power of discrimination

^d^Power of exclusion

**Fig. 1:**
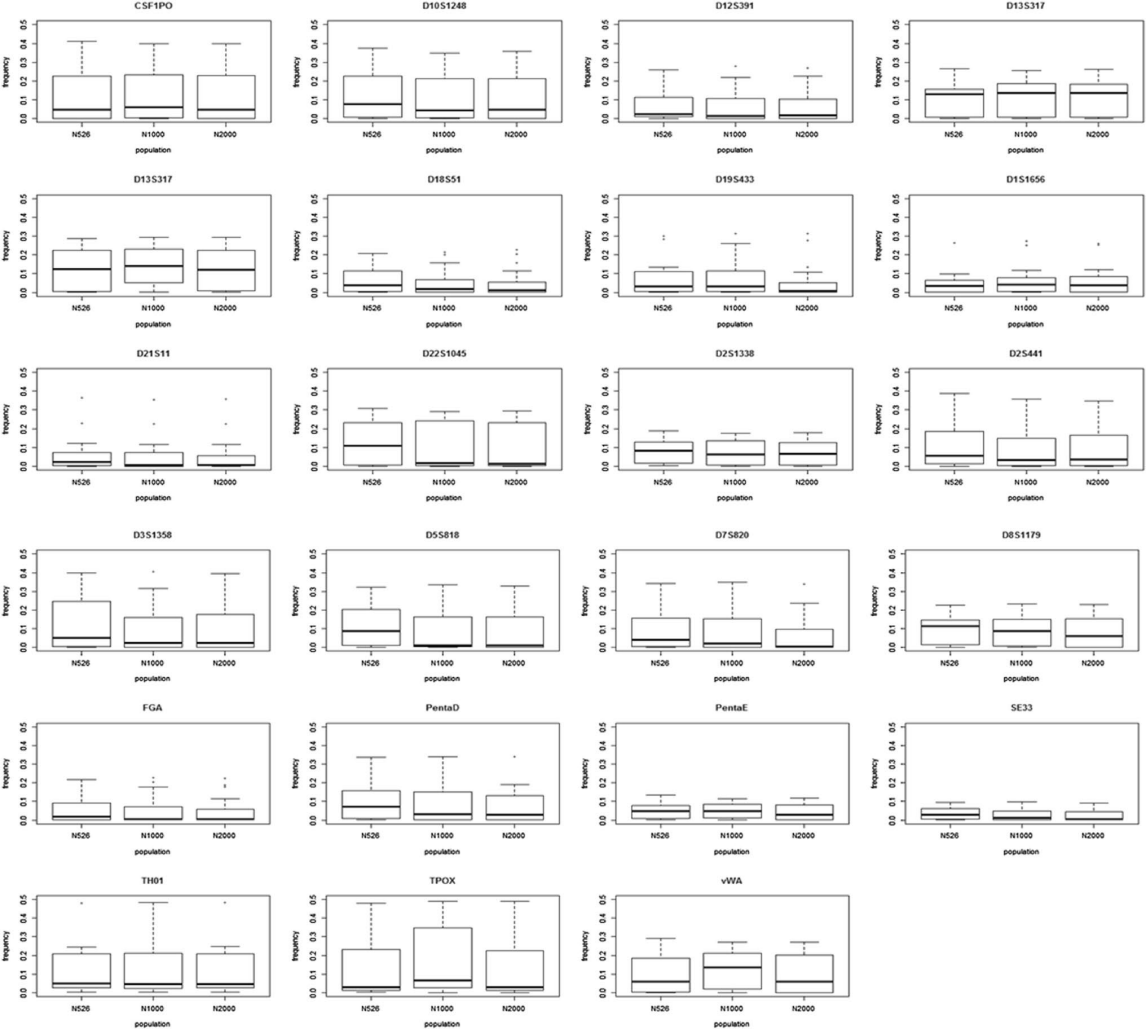
Range for allele frequencies of 23 loci

**Fig. 2:**
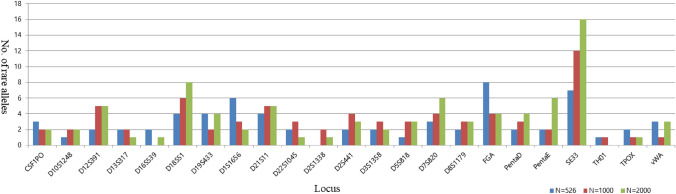
Comparison of the number of rare alleles in each locus

When comparing to allele frequency among three groups through the boxplot, there was a difference in the maximum allele frequency in the D19S433, PentaD, and TH01 loci. Particularly, the median values in the D22S1045 and D5S818 loci of group A and the vWA locus of group B were the highest. Allele frequencies in the D18S51, D19S433, and FGA loci had more outliers when the sample size was larger. In the TPOX locus, although the median of the allele frequency was similar among the three groups, the IQR was the widest in group B (Fig. 1). Many rare alleles in the D18S51, D7S820, Penta D, Penta E, and SE33 loci were observed in group C. Moreover, in group A, a relatively large number of rare alleles were observed in in the D1S1656 and FGA loci (Fig. 2). In particular, more rare alleles were observed in the SE33 locus that had the highest power of discrimination (PD) and power of exclusion (PE) (Table 1). Although many rare alleles were found in group B and group C, they were more frequently observed in a specific locus (e.g., D1S1656, FGA) of group A. It is considered to be an effect by sampling bias.

Notably, in the D2S441 locus, the allele of 9.1 had high frequencies of 0.044 and 0.049 in group B and group C, respectively, whereas it was not observed in group A (bold in Table 2). This phenomenon can be regarded as the following two cases. Firstly, this may be attributed to the sampling bias in Group A. As previous mentioned, as the sample size increases, the more allele variations such as rare alleles are observed. However, as shown in Fig. 2, observed numbers of the rare allele are not constant for each locus regardless of the sample size by sampling bias. Secondly, since different multiplex kits have been used for each population study, it may be affected by dropout of specific variant allele due to primer. D2S441 of Group A has been analyzed using the AmpF*l*STR™ NGM™ PCR amplification kit (NGM kit; Applied Biosystems, USA), and that of Group B and Group C has been analyzed using the GlobalFiler™ PCR Amplification Kit (GF; Applied Biosystems, USA) and PowerPlex^®^ Fusion system (PPF; Promega, USA). In the early NGM kit, dropout of population-specific variant allele was found in amelogenin, D2S441, and D22S1045 loci (Green et al. 2013). According to GF user guide, the allele of 9.1 in D2S441is an allele variant mainly found in Asian. Therefore, this observation may be the result by primer of multiplex kit that could not recover these specific variant alleles.

**Table 2:** Allele frequency of D2S441 locus in three sample groups

Locus	Allele	*N* = 526 (Group A)		*N* = 1,000 (Group B)		*N* = 2,000 (Group C)	
		AF^a^	OA^b^	AF	OA	AF	OA
D2S441	9	0.001	1	0.001	2	0.0005	2
	**9.1**	**ND**	**ND**	**0.044**	**88**	**0.0495**	**198**
	9.3	0.001	1	0.0005	1	0.0003	1
	10	0.185	195	0.21	420	0.2085	834

^a^Allele frequency

^b^Number of observed alleles

Sampling bias can affect the allele variation and the allele frequencies at specific loci. Several studies have reported that sample selection bias can affect population studies, such as ethnic group classification and ancestry inference (Shringarpure and Xing 2014; Risso et al. 2015). Even if the samples are derived from the same population, allele frequency and rare alleles can be affected by sample size, sampling bias, and heterozygosity ratio when performing population study. Moreover, because the MAF is useful in small-sized databases, it is necessary to obtain possible rare alleles within the population (Budowle et al. 1996). Restrepo et al. (2011) reported that the number of alleles with MAF increased in a large sample and the number of alleles with a constant frequency did not significantly change. In addition, the STR multiplex kit is also an important factor to study population variation. Several studies have described the null allele at specific locus or the discordance between multiplex kits (Mizuno et al. 2008; Tsuji et al. 2010; Raziel et al. 2012). Because the rare allele is corrected by 5/2 N when the probabilities were calculated, it does not have a significant effect between three groups on probability calculation such as likelihood ratio and random match probability. However, due to dropout of specific allele (in this study, allele dropout of 9.1 in the D2S441 locus of group A) with relatively high frequency, the calculation can lead to different results such as a difference of exponent in the likelihood ratio. For example, in Table 2, assuming that D2S441 allele 9.1 of individual A is homozygote, RMP is calculated as *p*^2^ (*p* is frequency of allele 9.1) and LR is calculated as 1/RMP. As a result, the RMPs of group B and group C are 1.9 × 10^–3^ and 2.4 × 10^–3^, respectively. However, since allele 9.1 of group A is dropout, so MAF (0.00475) is applied, and RMP of group A is 2.2 × 10^–5^. Furthermore, LR is 5.1 × 10^2^ and 4.1 × 10^2^ for group B and group C, and 4.5 × 10^4^ for group A. This may be statistically misinterpreted because the probability of coincidence is higher in group A. Therefore, it is necessary to use various multiplex kits for confirming concordance of allele.

In generally, the best way for reducing sampling bias is to obtain a large number of samples as possible. However, there is a limit to obtain many samples in practice. Therefore, it is necessary to make a sample selection utilizing auxiliary information such as region, age, sex and clan village (Shringapure and Xing 2014). Another way is to utilize the DNA database that contains the DNA profiles of many criminals. In Korea, the DNA database has about a hundred thousand DNA profiles of unrelated person. However, their use is strictly restricted by law. If it could be used only for allele frequency calculation, it would be of great help to forensic-related organizations and laboratories of Korea. Because the allele frequencies play an important role for probabilities in forensic genetics, it is important to consider the effects of sample size, sampling bias, and selection of multiplex kits in population studies.
